# Comparative effectiveness and integrated safety of goserelin sustained-release microspheres versus implants in prostate cancer: a patient-based real-world study and systematic review with meta-analysis

**DOI:** 10.3389/fonc.2026.1858453

**Published:** 2026-06-17

**Authors:** Jie Zhao, Qiang Zhao, Xiaohong Liu, Kun Hou, Yuan Chen, Hua Lan, Yaodong Ping

**Affiliations:** 1 School of Pharmacy, Inner Mongolia Medical University, Hohhot, China; 2 Department of Pharmacy, Peking University Cancer Hospital Inner Mongolia Hospital, Hohhot, China; 3 Key Laboratory of Carcinogenesis and Translational Research (Ministry of Education/Beijing), Department of Urology, Peking University Cancer Hospital & Institute, Beijing, China; 4 Key Laboratory of Carcinogenesis and Translational Research (Ministry of Education/Beijing), Department of Pharmacy, Peking University Cancer Hospital & Institute, Beijing, China

**Keywords:** androgen deprivation therapy, goserelin sustained-release implant, goserelin sustained-release microspheres, meta-analysis, prostate cancer, prostate-specific antigen, real-world study, safety

## Abstract

**Objective:**

To evaluate the effectiveness of goserelin sustained-release microspheres versus sustained-release implants for androgen deprivation therapy (ADT) in patients with prostate cancer in a real-world setting, and to compare their safety profiles by integrating evidence from published studies.

**Methods:**

This retrospective study included 87 patients with prostate cancer who received goserelin-based ADT at Peking University Cancer Hospital Inner Mongolia Hospital between August 2024 and September 2025. Forty-four patients received goserelin sustained-release microspheres, and 43 received sustained-release implants. During the 12-week observation period, patients received median administrations of three and one, respectively, corresponding to comparable ADT exposure. Analyses were patient-based. The primary endpoint was the proportion achieving total prostate-specific antigen (TPSA) <0.2 ng/mL at day 85 (D85), with day 29 (D29) assessed as an early response point. A prespecified exploratory non-inferiority framework with a −10% margin was applied, with sensitivity analyses using TPSA <0.1 ng/mL. A PRISMA-compliant systematic review searched PubMed, Embase, Cochrane Library, and Web of Science through February 1, 2026. Eligible studies reporting goserelin safety outcomes were included. Safety outcomes were harmonized as any-grade adverse events (AEs), serious AEs, and grade ≥3 AEs. Pooled incidences were calculated using single-arm meta-analysis.

**Results:**

Median age was 72 years, and median follow-up was 85 days. Baseline disease stage differed between groups, with more metastatic disease in the implant group. At D29, TPSA response rates were 81.8% and 95.2% in the microsphere and implant groups, respectively. At D85, rates were 86.4% and 93.0%, meeting the prespecified non-inferiority criterion. Sensitivity analyses using TPSA <0.1 ng/mL were consistent. The systematic review included three studies comprising 665 patients. The pooled incidence of any-grade AEs was 59.2%, with substantial heterogeneity, whereas pooled incidences of serious AEs and grade ≥3 AEs were 6.1% and 8.9%, respectively, with no new safety signals.

**Conclusions:**

Goserelin sustained-release microspheres demonstrated comparable real-world biochemical activity to sustained-release implants over matched 12-week ADT exposure. Although early PSA differences and baseline disease-stage imbalance warrant consideration, integrated evidence supports goserelin microspheres as an alternative ADT formulation with a manageable safety profile.

**Systematic review registration:**

https://www.crd.york.ac.uk/PROSPERO/view/, identifier CRD420261327915.

## 1 Introduction

Prostate cancer (PCa) is the second most commonly diagnosed malignancy among men worldwide and represents a significant global health burden (1). Its incidence increases markedly with age and is higher in developed regions (2). Androgen deprivation therapy (ADT) remains the cornerstone of treatment for advanced disease, aiming to suppress serum testosterone to castration levels and thereby inhibit tumor progression (3). This is primarily achieved using gonadotropin-releasing hormone (GnRH) agonists or antagonists, which act through the hypothalamic–pituitary–gonadal axis (4, 5).

Prostate-specific antigen (PSA) is the most widely used biomarker for the diagnosis and therapeutic monitoring of PCa (6). Accumulating evidence suggests that changes in PSA levels reflect tumor burden and treatment response. In the early phase of ADT, both the rate of PSA decline and the achievement of predefined PSA thresholds have been associated with disease control and clinical outcomes (7–9). Accordingly, PSA-based endpoints have been widely used to evaluate the effectiveness of GnRH agonists in patients with PCa.

Goserelin, a widely used GnRH agonist, is available in two formulations: a sustained-release implant and sustained-release microspheres. Although these formulations share the same pharmacological mechanism, they differ in administration routes, drug release profiles, and potential impacts on treatment adherence. Previous studies have suggested that goserelin formulations are effective in achieving testosterone suppression and reducing PSA levels (10). However, direct comparative evidence between goserelin sustained-release microspheres and implants in real-world clinical practice remains limited. Although comparative reviews of GnRH agonists have been published (11, 12), no prior systematic review or meta-analysis has specifically compared the safety and effectiveness of goserelin sustained-release microspheres versus implants in patients with PCa.

Therefore, this study aimed to compare the effectiveness and safety of goserelin sustained-release microspheres and implants in patients with PCa, using PSA as the primary endpoint. To address the limited sample size for safety evaluation in the real-world cohort, evidence from published studies was integrated through meta-analysis to enhance the assessment of adverse events. This integrated approach provides comparative evidence to support formulation selection in clinical practice.

## 2 Methods

### 2.1 Study design

This study comprised two complementary components: a patient-based retrospective real-world comparative analysis and a systematic review with single-arm meta-analysis of safety outcomes. The real-world analysis compared PSA suppression between patients receiving goserelin sustained-release microspheres and those receiving sustained-release implants. The systematic review and meta-analysis were conducted to provide a broader assessment of safety outcomes, particularly given the limited sample size of the real-world cohort for detecting infrequent adverse events.

The systematic review was conducted in accordance with the PRISMA guidelines, and the completed PRISMA checklist is provided in **Supplementary Table 1**. The study protocol was registered in PROSPERO (CRD420261327915).

### 2.2 Real-world study population

#### 2.2.1 Participants

Patients with histologically confirmed PCa who received goserelin-based ADT at Peking University Cancer Hospital Inner Mongolia Hospital between August 2024 and September 2025 were retrospectively included. The analysis was conducted on a patient basis rather than a dose basis. Each patient was treated as a single analytical unit and contributed one set of baseline characteristics and one PSA assessment at each scheduled follow-up time point.

To ensure comparable androgen deprivation exposure between formulations, patients receiving microspheres generally underwent three consecutive 3.6 mg administrations every 4 weeks, whereas patients receiving implants received one 10.8 mg administration covering the same 12-week treatment duration. This exposure equivalence has been supported by previous pharmacodynamic studies demonstrating that one 10.8 mg depot is comparable to three successive 3.6 mg depots (13).

Inclusion criteria were as follows: male patients aged ≥18 years; histologically confirmed prostate cancer based on prostate biopsy; availability of imaging data for disease staging; and receipt of goserelin-based ADT for at least 3 months. Exclusion criteria included prior or ongoing endocrine therapy for prostate cancer, castration-resistant prostate cancer, history of pituitary or adrenal surgery, known drug hypersensitivity, or participation in other clinical studies.

This study was approved by the institutional ethics committee of Peking University Cancer Hospital Inner Mongolia Hospital (No. KY202543). Written informed consent was obtained from all patients.

#### 2.2.2 Treatment and follow-up

A total of 44 patients in the experimental group received goserelin microspheres (3.6 mg every 4 weeks), while 43 patients in the control group received sustained-release implants (10.8 mg every 12 weeks), as part of ADT.

Clinical data were collected, including demographic characteristics, Eastern Cooperative Oncology Group (ECOG) performance status, disease status, prior treatments, surgical history, and serum PSA levels at baseline, Day 29, and Day 85.

#### 2.2.3 Statistical analysis

A non-inferiority design was applied, with the PSA response rate defined as the primary endpoint to compare the microsphere group and the sustained-release implant group.

Secondary endpoints included the rate of deep PSA suppression (PSA <0.1 ng/mL), the proportion of patients achieving FPSA <0.01 ng/mL, and the distribution of continuous PSA and FPSA levels at different time points.

Sample size was calculated based on a non-inferiority test for the difference between two proportions. Assuming a PSA response rate of 90% in both groups, with a one-sided significance level of α = 0.025 and a statistical power of 1 − β = 0.90, at least 23 patients were required per group. Considering an anticipated 20% loss to follow-up, a minimum of 29 patients per group was required. A total of 87 patients were ultimately included, meeting the predefined sample size requirements.

A non-inferiority margin of −10% was prespecified for the primary endpoint. This margin was selected based on clinical considerations and consistency with previously reported non-inferiority studies evaluating androgen deprivation therapies and PSA response outcomes in prostate cancer treatment (11, 12). Considering the exploratory nature of this real-world study and the expected variability in PSA responses across clinical practice settings, a 10% margin was considered clinically acceptable to determine whether goserelin sustained-release microspheres retained comparable biochemical efficacy relative to sustained-release implants.

All statistical analyses were performed using R software (version 4.5.2). Continuous variables are presented as median and interquartile range (IQR), and categorical variables as percentages.

### 2.3 Integrated safety analysis

To complement the limited sample size of the real-world cohort in safety evaluation, a systematic literature review and single-arm meta-analysis were conducted to provide an integrated assessment of adverse events associated with goserelin.

The safety outcomes of interest were any-grade AEs, SAEs, and grade ≥3 AEs. Safety outcomes were extracted according to the definitions reported in each original study. When toxicity grading criteria were available, CTCAE or CTCAE-compatible grading was used. When CTCAE grading was not explicitly reported, investigator-reported AE and SAE categories were retained. Reference standards for adverse event ascertainment were categorized as biochemical, clinical, imaging-based, or multimodal assessments according to the information provided in each study.

Eligible studies included randomized controlled trials (RCTs) and non-randomized interventional studies involving patients with histologically confirmed prostate cancer who received goserelin treatment and reported safety outcomes. Studies were excluded if safety data were not reported, if they were duplicate publications, or if full texts or extractable data were unavailable.

A systematic literature search was conducted in PubMed, Embase, the Cochrane Library, and Web of Science from database inception to February 1, 2026. Both controlled vocabulary and free-text terms were used, including “prostate cancer” and “goserelin.” In addition, reference lists of relevant articles were screened to identify additional studies.

Two reviewers independently screened the literature, with initial screening based on titles and abstracts, followed by full-text review for eligibility. Data extraction was performed independently by two reviewers, including author, publication year, country, study design, intervention dose and follow-up duration, patient characteristics, and safety outcomes. Risk of bias was assessed using the RoB 2 and ROBINS-I tools by two independent reviewers, with disagreements resolved through discussion.

A single-arm meta-analysis was performed to pool the incidence of adverse events. To stabilize variance, raw proportions were transformed using the logit transformation (PLOGIT). A continuity correction of 0.5 was applied to studies with zero events or events equal to the total sample size.

A random-effects model was used to estimate pooled effects, with between-study heterogeneity (τ²) estimated using a generalized linear mixed model (GLMM). The Hartung–Knapp method was applied to calculate pooled estimates and 95% confidence intervals.

Heterogeneity was assessed using Cochran’s Q test and the I² statistic. Subgroup analyses by formulation type and dosage, as well as leave-one-out sensitivity analyses, were performed. Publication bias was assessed using funnel plots.

All analyses were conducted in R (version 4.5.2), primarily using the meta and dplyr packages.

## 3 Results

### 3.1 Real-world study results

A total of 87 patients with PCa were included in the real-world cohort, including 44 patients who received goserelin sustained-release microspheres and 43 patients who received goserelin sustained-release implants. The overall median age was 72 years, and the median follow-up duration was 85 days.

Baseline characteristics were generally similar between groups with respect to age, Gleason grade, ECOG performance status, and most prior treatment variables. However, disease-stage distribution was imbalanced. The implant group included a higher proportion of patients with metastatic disease than the microsphere group, whereas localized disease was more frequent in the microsphere group. In addition, prior radiotherapy was more common in the implant group. Baseline demographic and clinical characteristics are summarized in **Table 1**.

**Table 1:** Baseline demographic and clinical characteristics of patients with prostate cancer (n = 87).

Characteristics	Microsphere (n = 44)	Implant (n = 43)	P value
Median age (years)	71 (55, 90)	73 (55, 88)	0.1289
Stage of disease, n (%)			
Localized	13 (29.5)	1 (2.3)	0.0244
Locally advanced	7 (15.9)	4 (9.3)	0.5378
Metastatic	18 (40.9)	33 (76.7)	0.1206
None	6 (13.6)	5 (11.6)	0.8514
Gleason grade, n (%)			
6	2 (4.5)	2 (4.7)	0.9862
7	9 (20.5)	9 (20.9)	0.9724
8	12 (27.3)	10 (23.3)	0.791
9-10	12 (27.3)	10 (23.3)	0.791
Missing	9 (20.5)	12 (27.9)	0.6149
ECOG performance status, n (%)			
0	34 (77.3)	28 (65.1)	0.6329
1	10 (22.7)	15 (34.9)	0.452
Prior anticancer therapy, n (%)			
Yes	19 (43.2)	10 (23.3)	0.2524
No	25 (56.8)	33 (76.7)	0.4183
Type of prior therapy, n (%)			
Radiotherapy	8 (18.2)	21 (48.8)	0.0783
Chemotherapy	5 (11.4)	5 (11.6)	0.9789
None	31 (70.5)	17 (39.5)	0.1674

Data are presented as median (range) or n (%). ECOG, Eastern Cooperative Oncology Group; n, number of patients. P values were calculated using the Wilcoxon rank-sum test for continuous variables and the chi-square test or Fisher’s exact test for categorical variables, as appropriate. P values are descriptive because of the retrospective study design and limited sample size.

**Figure 1:**
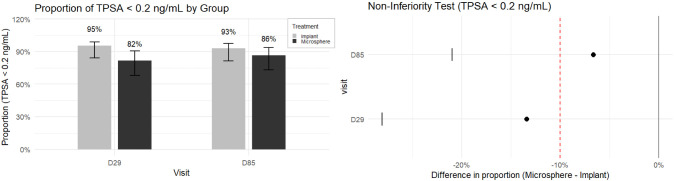
Proportion of patients achieving serum TPSA < 0.2 ng/mL at D29 and D85, with non-inferiority analysis between treatment groups.

At D29, the proportions of patients achieving serum PSA < 0.2 ng/mL were 82% in the microsphere group and 95% in the implant group; at D85, the corresponding rates were 86% and 93%, respectively (**Figure 1**). The between-group differences in response rates (microspheres minus implants) were within the prespecified non-inferiority margin of −10%, with the lower bounds of the 95% confidence intervals exceeding this threshold at both time points.

**Figure 2:**
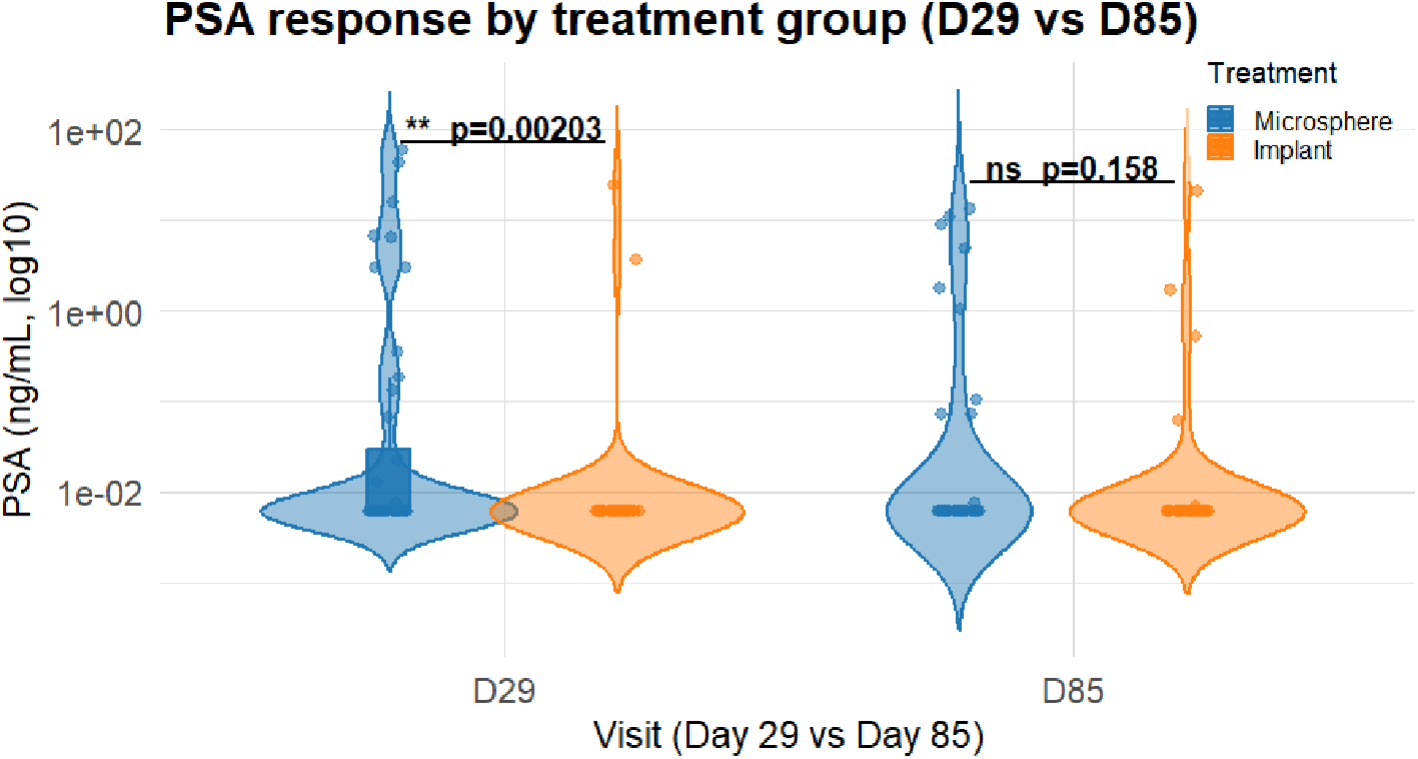
Distribution of serum TPSA levels at D29 and D85 across treatment groups. **p=0.00203; ns, p=0.158.

Analysis of log_10_-transformed continuous serum PSA levels (**Figure 2**) showed that at D29, the microsphere group had a slightly higher median and a wider distribution. By D85, the medians and distributions of the two groups largely overlapped, with reduced between-group differences over time.

**Figure 3:**
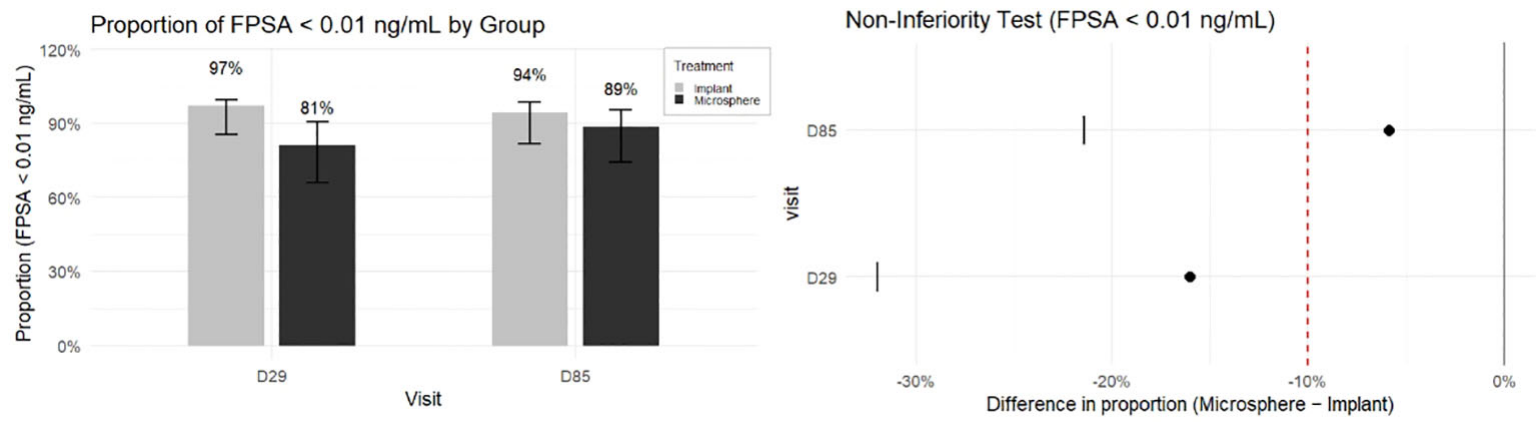
Proportion of patients achieving serum FPSA < 0.01 ng/mL at D29 and D85, with non-inferiority analysis.

At D29, the proportions of patients achieving FPSA < 0.01 ng/mL were 81% in the microsphere group and 97% in the implant group, increasing to 89% and 94% at D85, respectively (**Figure 3**). The between-group differences were within the prespecified non-inferiority margin of −10%, with the lower bounds of the 95% confidence intervals exceeding this threshold at both time points.

**Figure 4:**
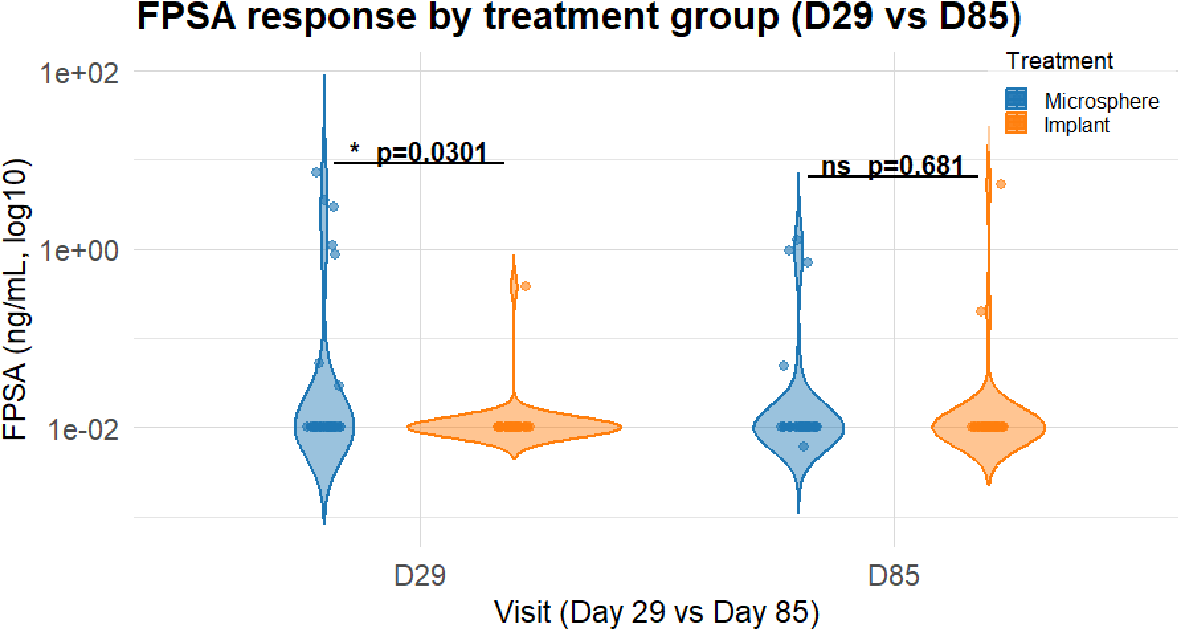
Distribution of serum FPSA levels at D29 and D85 across treatment groups. *p=0.0301; ns, p=0.681.

Analysis of continuous FPSA distributions (**Figure 4**) showed greater inter-individual variability in the microsphere group at D29. By D85, the medians and distribution ranges of the two groups largely overlapped.

Sensitivity analyses (**Table 2**), using a more stringent PSA threshold (< 0.1 ng/mL) and alternative handling of values below the limit of detection, showed no substantial changes in the direction or magnitude of between-group differences.

**Table 2:** Sensitivity analyses of non-inferiority for PSA outcomes.

Visit	Marker	Analysis	Microsphere	CI (microsphere)	Implant	CI (implant)	Difference
D29	TPSA	Main (TPSA < 0.2)	36/44 (81.8%)	67.3%, 91.8%	40/42 (95.2%)	83.8%, 99.4%	-0.134
D85	TPSA	Main (TPSA < 0.2)	38/44 (86.4%)	72.6%, 94.8%	40/43 (93.0%)	80.9%, 98.5%	-0.067
D29	TPSA	Sensitivity (TPSA < 0.1)	34/44 (77.3%)	62.2%, 88.5%	40/42 (95.2%)	83.8%, 99.4%	-0.18
D85	TPSA	Sensitivity (TPSA < 0.1)	37/44 (84.1%)	69.9%, 93.4%	40/43 (93.0%)	80.9%, 98.5%	-0.089
D29	FPSA	Main (FPSA < 0.01)	30/37 (81.1%)	64.8%, 92.0%	34/35 (97.1%)	85.1%, 99.9%	-0.161
D85	FPSA	Main (FPSA < 0.01)	31/35 (88.6%)	73.3%, 96.8%	34/36 (94.4%)	81.3%, 99.3%	-0.059
D29	FPSA	Sensitivity (FPSA < 0.005)	0/37 (0.0%)	0.0%, 9.5%	0/35 (0.0%)	0.0%, 10.0%	0
D85	FPSA	Sensitivity (FPSA < 0.005)	0/35 (0.0%)	0.0%, 10.0%	0/36 (0.0%)	0.0%, 9.7%	0

CI, confidence interval; D, day; FPSA, free prostate-specific antigen; TPSA, total prostate-specific antigen. Data are presented as n/N (%), where n represents the number of responders and N represents the total number of evaluable patients. “Main” indicates the primary analysis using predefined thresholds, whereas “Sensitivity” indicates sensitivity analyses using more stringent thresholds or alternative handling of values below the limit of detection. The Clopper–Pearson method was used to calculate 95% confidence intervals. Difference refers to the response rate difference between the Microsphere group and the Implant group (Microsphere minus Implant).

### 3.2 Meta-analysis results

#### 3.2.1 Study selection and quality assessment

**Figure 5:**
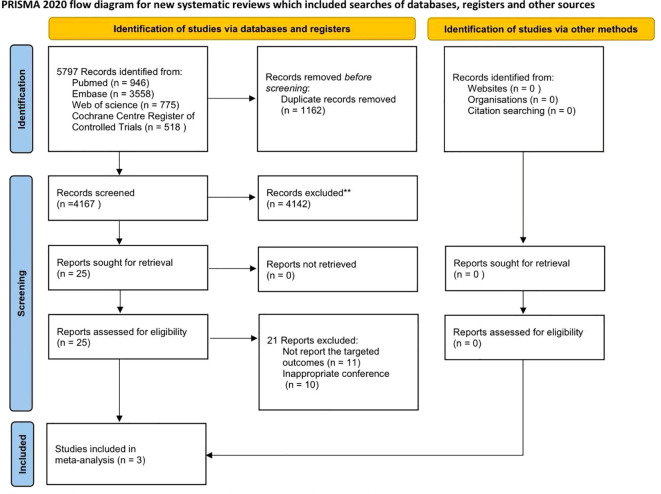
PRISMA flow diagram illustrating the study selection process.

A total of 5,794 records were identified through database searching (**Figure 5**). After duplicate removal and screening of titles and abstracts, full texts of potentially eligible studies were assessed. Ultimately, three studies involving 665 patients were included in the safety meta-analysis: Gu et al. (10), Dijkman et al. (13), and Chen et al. (14).The included studies comprised two randomized controlled trials and one prospective cohort study. The interventions included goserelin sustained-release microspheres (3.6 mg) and sustained-release implants (3.6 mg or 10.8 mg). Study populations included patients with localized, locally advanced, or metastatic prostate cancer, with follow-up durations ranging from 12 to 48 weeks. Study characteristics are summarized in **Table 3**.

**Table 3:** Characteristics of studies included in the meta-analysis.

ArmID	StudyID	Study year	Country	Intervention	Dose	Design	Population	N	Any AE event	AE definition	Grade 3 plus AE event	InjSite AE event	Serious AE event	Follow up weeks
Arm1	LY01005_ RCT	2023	China	Microsphere	3.6mg	RCT	localized; locally advanced prostate cancer; Metastatic	144	117	TEAE	10	0	7	12
Arm2	LY01005_ RCT	2023	China	Implant	3.6mg	RCT	localized; locally advanced prostate cancer; Metastatic	145	124	TEAE	11	1	5	12
Arm3	GOS_ RWE_108	2024	China	Implant	10.8mg	Prospective cohort	localized; locally advanced prostate cancer	294	117	TEAE	31	NA	30	26
Arm4	ZOLA_ RCT	1995	Netherlands	Implant	10.8mg	RCT	advanced prostatic carcer	39	12	AE	NA	NA	NA	48
Arm5	ZOLA_ RCT	1995	Netherlands	Implant	3.6mg	RCT	advanced prostatic carcer	43	19	AE	NA	NA	NA	48

AE, adverse event; SAE, serious adverse event; TEAE, treatment-emergent adverse event; RCT, randomized controlled trial; NA, not available. The included studies used different methods and terminology for adverse event reporting. Gu et al. and Chen et al. reported treatment-emergent adverse events (TEAEs) and serious adverse events (SAEs), whereas Dijkman et al. did not explicitly describe the adverse event assessment criteria or reporting standard. Because adverse event definitions and reporting methods were not fully standardized across studies, pooled safety estimates should be interpreted with caution.

Risk of bias was assessed using the Cochrane Risk of Bias tool (RoB 2.0) for the two randomized controlled trials (10, 13), both of which were judged to be at low risk of bias across all domains. The cohort study (14) was also assessed using the ROBINS-I tool and was judged to be at low risk of bias. Detailed results are presented in **Supplementary Figures 1**, **2**.

#### 3.2.2 Single-arm meta-analysis results

**Figure 6:**
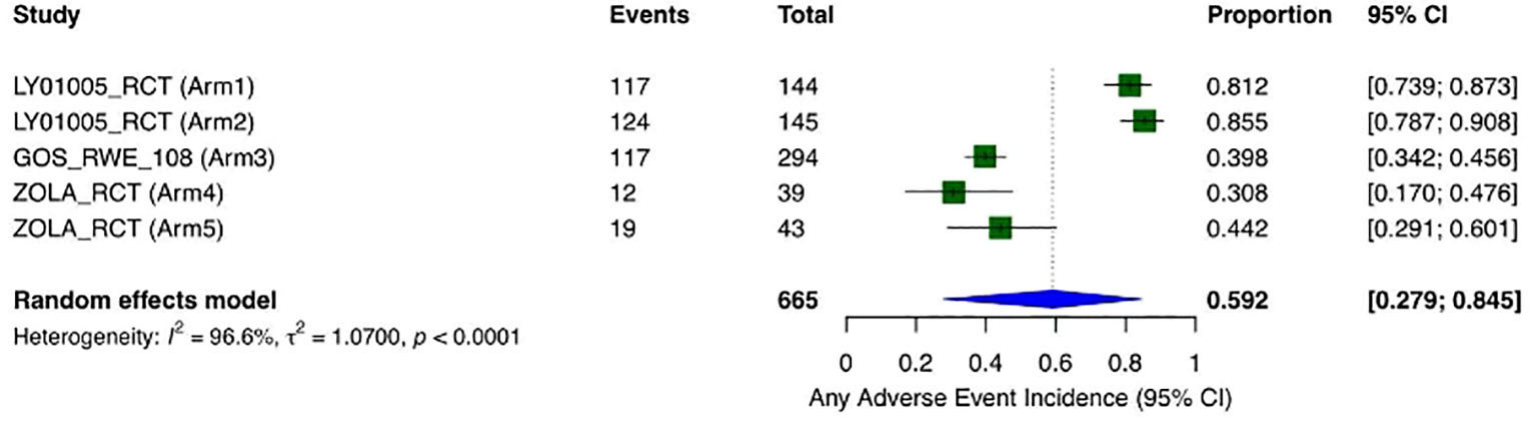
Forest plot of the pooled incidence of adverse events of any grade.

The pooled incidence of adverse events of any grade was 59.2% (95% CI: 27.9%–84.5%), with substantial heterogeneity (I² = 96.6%) (**Figure 6**). Adverse events were extracted according to the safety reporting methods described in the original studies. However, the definitions and ascertainment methods of adverse events were not fully standardized across studies.

**Figure 7:**
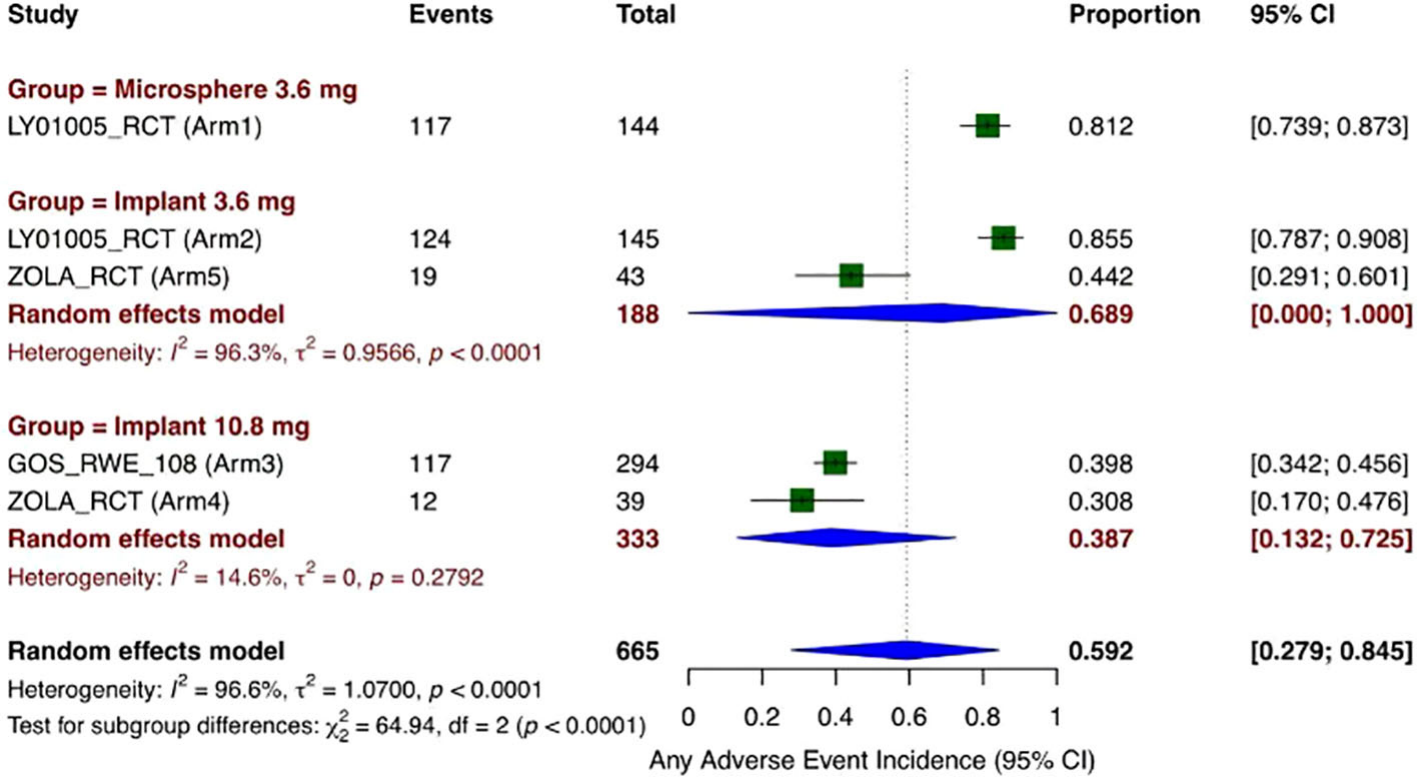
Subgroup analysis of adverse event incidence by formulation type and dosage.

Subgroup analysis by formulation and dose (**Figure 7**) revealed a higher incidence in the 3.6 mg microsphere group. The 10.8 mg implant group was associated with a lower incidence and minimal heterogeneity (I² = 14.6%). The subgroup difference was statistically significant (P < 0.0001).

**Figure 8:**
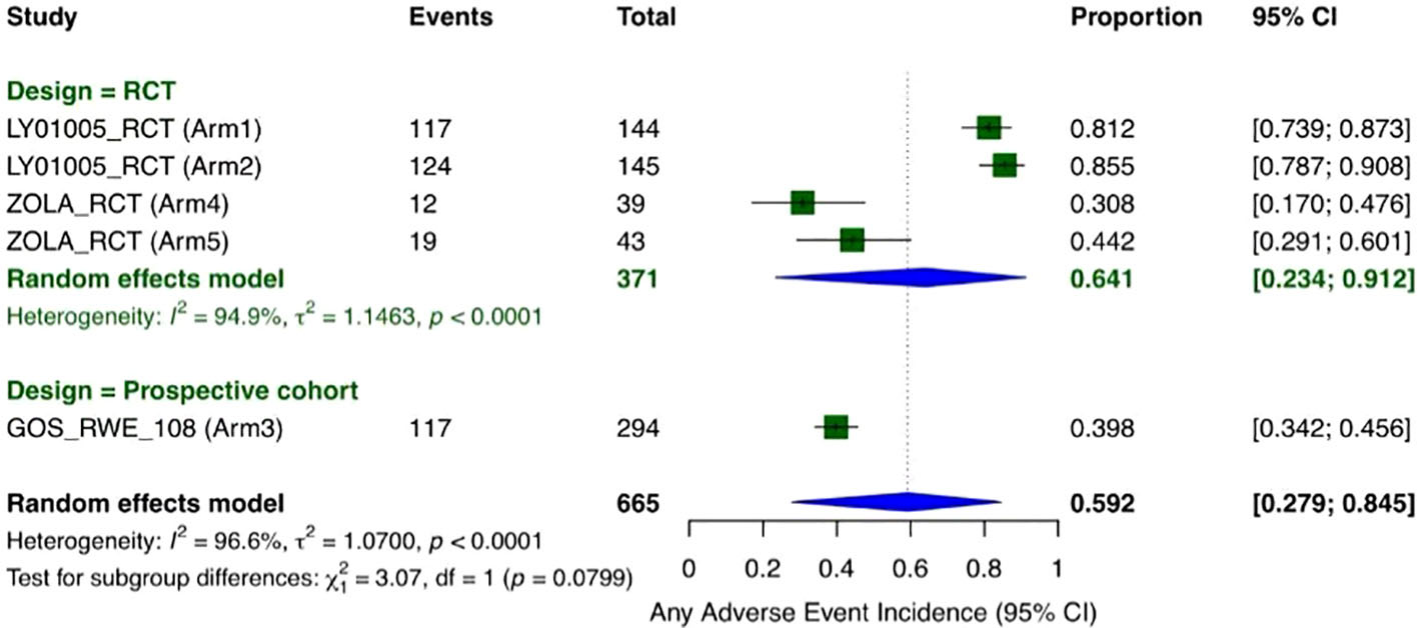
Subgroup analysis of adverse event incidence by study design.

Subgroup analysis by study design (**Figure 8**) demonstrated a higher incidence of adverse events in randomized controlled trials than in the prospective cohort study. The between-group difference did not reach statistical significance (P = 0.0799).

**Figure 9:**
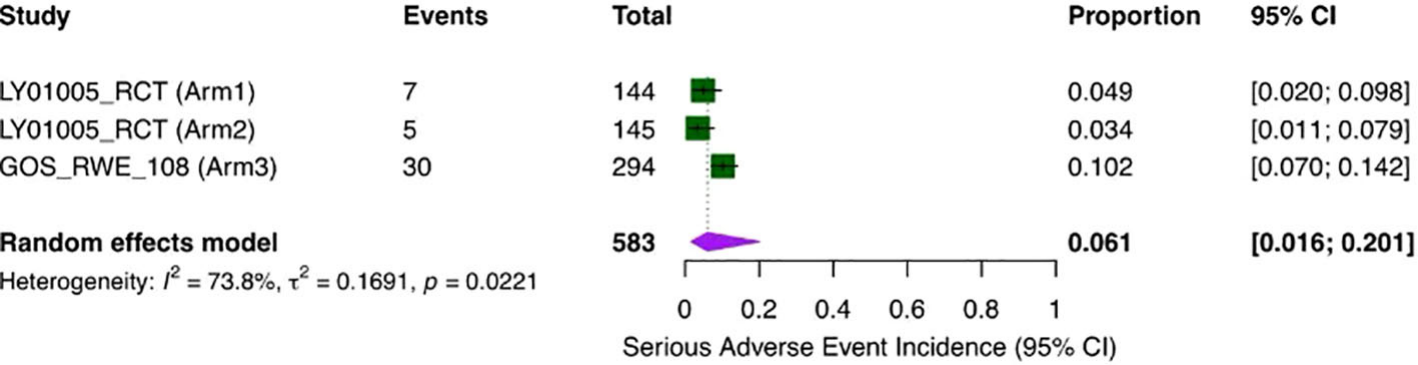
Forest plot of the pooled incidence of serious adverse events.

**Figure 10:**
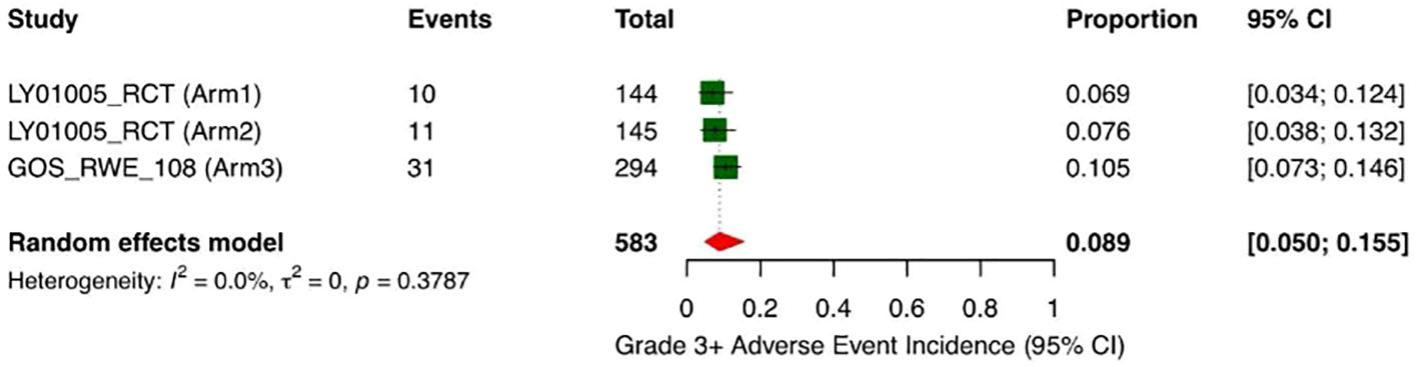
Forest plot of the pooled incidence of grade ≥3 adverse events.

The pooled incidence of serious adverse events was 6.1% (95% CI: 1.6%–20.1%; I² = 73.8%) (**Figure 9**). The incidence of grade ≥3 adverse events was 8.9% (95% CI: 5.0%–15.5%; I² = 0%) (**Figure 10**).

**Figure 11:**
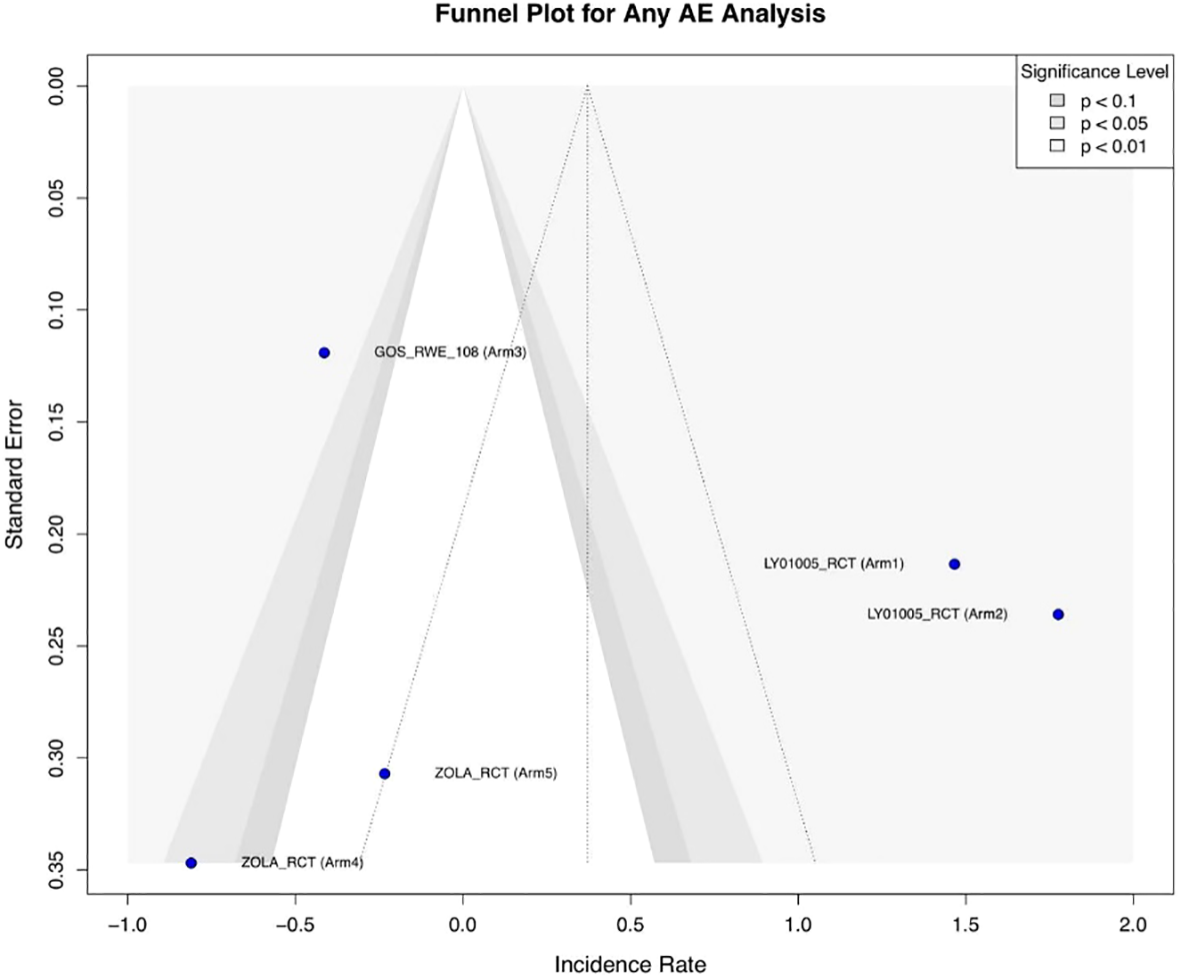
Funnel plot assessing publication bias.

**Figure 12:**
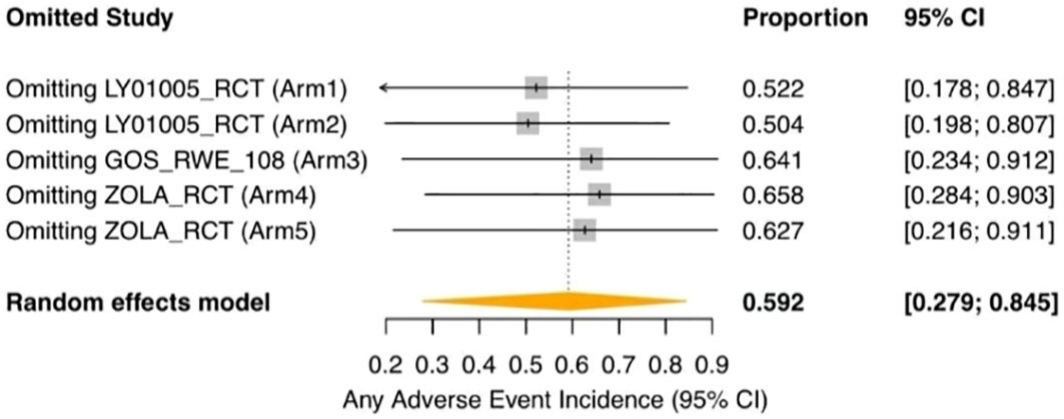
Sensitivity analysis of pooled adverse event outcomes.

Assessment of publication bias (**Figure 11**) revealed asymmetry in the funnel plot. Sensitivity analysis (**Figure 12**) indicated that sequential exclusion of individual studies did not materially alter the pooled estimates.

By integrating real-world data with evidence from the literature, this study demonstrates that goserelin microspheres are non-inferior to sustained-release implants in achieving PSA suppression in patients with prostate cancer undergoing ADT, within the predefined non-inferiority margin.

Based on the meta-analysis, the overall safety profiles of the two formulations are comparable, with a low incidence of serious adverse events. However, these findings should be interpreted with caution given the limited available evidence.

Overall, goserelin microspheres may represent a viable alternative to sustained-release implants in clinical practice.

## 4 Discussion

### 4.1 Efficacy

This study demonstrated that goserelin sustained-release microspheres achieved non-inferior biochemical suppression compared with sustained-release implants at D85, with comparable PSA and FPSA response rates and similar distributions of continuous measures. These findings support the clinical comparability of the two formulations and are consistent with previous evidence showing minimal differences in long-term efficacy among GnRH agonists (11, 12).

A time-dependent pattern in PSA dynamics was observed. At D29, the microsphere group showed lower response rates, higher median PSA levels, and greater inter-individual variability; however, these differences diminished over time, with convergence of outcomes by D85. This suggests that early PSA differences primarily reflect variations in the onset of treatment response rather than overall efficacy.

These observations can be partly attributed to differences in pharmacokinetic characteristics between formulations. Sustained-release microspheres release the drug gradually through polymer degradation, resulting in relatively stable plasma concentrations with reduced peak–trough fluctuations (15, 16), whereas sustained-release implants may achieve effective systemic exposure more rapidly after administration.

This difference is consistent with the slower decline in PSA observed in the microsphere group at D29, with the between-group disparity diminishing over time (16, 17). Variations in exposure kinetics during the early phase of treatment may influence the timing of testosterone suppression and, consequently, PSA dynamics (13, 18, 19). Previous studies have similarly reported differences in drug exposure profiles and time to steady state among GnRH agonist formulations (15, 16, 20).

Interpretation of the early treatment responses in this study should take into account the imbalance in baseline disease stage between the two groups. The implant group comprised a greater proportion of patients with metastatic disease, whereas localized disease was more prevalent in the microsphere group. Previous evidence suggests that patients with higher tumor burden or elevated baseline PSA levels may exhibit less rapid PSA responses following androgen deprivation therapy (18, 19, 21). Accordingly, this baseline imbalance may have influenced early PSA dynamics and contributed to variability in treatment responses during the initial treatment phase (22, 23). Moreover, drug exposure is closely associated with the magnitude and kinetics of PSA reduction (21, 24, 25). In real-world settings, variations in dosing schedules and treatment adherence may further amplify early PSA fluctuations (13, 18, 19). With continued treatment, pharmacological effects are likely to predominate, while the influence of baseline characteristics and external factors gradually attenuates.

From a clinical perspective, these findings indicate that goserelin microspheres represent a viable alternative to implants. Importantly, early PSA measurements should be interpreted cautiously, and treatment decisions should rely on longitudinal PSA dynamics rather than single early time points, and should not be driven solely by early differences between formulations (26, 27).

### 4.2 Safety

The integrated safety analysis indicates that safety outcomes were comparable between goserelin microspheres (3.6 mg) and sustained-release implants (3.6 mg). The pooled incidence of any-grade adverse events (AEs) was 59.2%, with substantial heterogeneity (I² = 96.6%). In contrast, the incidence of serious adverse events (SAEs; 6.1%) and grade ≥3 AEs (8.9%; I² = 0%) was low and consistent across studies, indicating a stable risk of severe toxicity.

Given the inherent limitation of sample size in real-world studies for detecting less frequent adverse events, the incorporation of this meta-analysis provides a more robust and comprehensive safety evaluation, complementing rather than substituting the findings from the real-world cohort.

This finding is consistent with the established safety profile of androgen deprivation therapy (ADT). Adverse events are primarily related to androgen suppression, including hot flashes, fatigue, and sexual dysfunction, whereas severe toxicities remain uncommon (28, 29). Given that both formulations share the same active ingredient and mechanism of action, clinically meaningful differences in safety profiles are not expected (12).

The substantial heterogeneity observed for any-grade adverse events is likely driven by methodological differences rather than true variations in treatment effects (30). Such differences include variations in monitoring intensity, reporting practices, and patient population characteristics. The higher incidence of adverse events reported in randomized controlled trials (RCTs) is consistent with previous evidence suggesting that more intensive monitoring may increase event detection rates (31).

The findings of the present analysis are consistent with previous studies (32–34). The incidence of serious adverse events (6.1%) is lower than that reported in earlier studies (approximately 11%), but remains within the reported range, supporting the conclusion that GnRH agonists are associated with a limited risk of severe toxicity. The potential differences observed in dose-stratified analyses warrant further investigation.

### 4.3 Limitations

Several limitations of this study should be acknowledged. First, this retrospective real-world analysis was conducted in a single-center cohort. The regional nature of the cohort and baseline imbalances in disease staging may limit the generalizability of the findings and potentially confound early PSA dynamics. Although this imbalance may reflect real-world treatment selection patterns, the absence of adjusted analyses may have introduced residual confounding.

Second, key pharmacodynamic biomarkers, particularly serum testosterone levels, were not available, which limits mechanistic interpretation of treatment effects. Since testosterone suppression represents the primary pharmacological mechanism of androgen deprivation therapy, the absence of testosterone monitoring limits mechanistic interpretation of PSA dynamics and treatment response. Nevertheless, PSA response remains a clinically relevant surrogate biomarker that has been widely used to assess treatment response and clinical outcomes in patients with prostate cancer (7–9).

Third, the safety meta-analysis included a limited number of studies and study arms. The included studies differed in design, follow-up duration, adverse-event definitions, toxicity grading, and monitoring intensity. These factors contributed to heterogeneity, especially for any-grade AEs, and limited the ability to perform detailed analyses of specific adverse events.

Finally, the relatively short follow-up duration precludes adequate assessment of long-term outcomes. Further large-scale, multicenter prospective studies are warranted to validate these findings.

Despite these limitations, this study integrates real-world efficacy data with systematically synthesized safety evidence, providing a comprehensive evaluation of goserelin formulations and supporting informed treatment selection in clinical practice.

## 5 Conclusion

In this patient-based real-world comparative study, goserelin sustained-release microspheres showed PSA suppression patterns broadly comparable to those of sustained-release implants over a 12-week ADT exposure period. Early PSA responses differed between formulations, but response rates and PSA distributions converged by D85. Integrated safety evidence further suggested comparable safety profiles, with a low incidence of severe adverse events. These findings support goserelin sustained-release microspheres as a clinically relevant alternative to sustained-release implants in patients with PCa. Further large-scale, multicenter prospective studies with standardized adverse-event reporting, testosterone monitoring, and longer follow-up are warranted to confirm these findings.

## Data Availability

The original contributions presented in the study are included in the article/**Supplementary Material**. Further inquiries can be directed to the corresponding author.
