# Supplementary figures and images for: Comparative effectiveness and integrated safety of goserelin sustained-release microspheres versus implants in prostate cancer: a patient-based real-world study and systematic review with meta-analysis
Source: Front Oncol. 2026 Jun 17;16:1858453. doi: 10.3389/fonc.2026.1858453 (PMC13318708; doi:10.3389/fonc.2026.1858453)

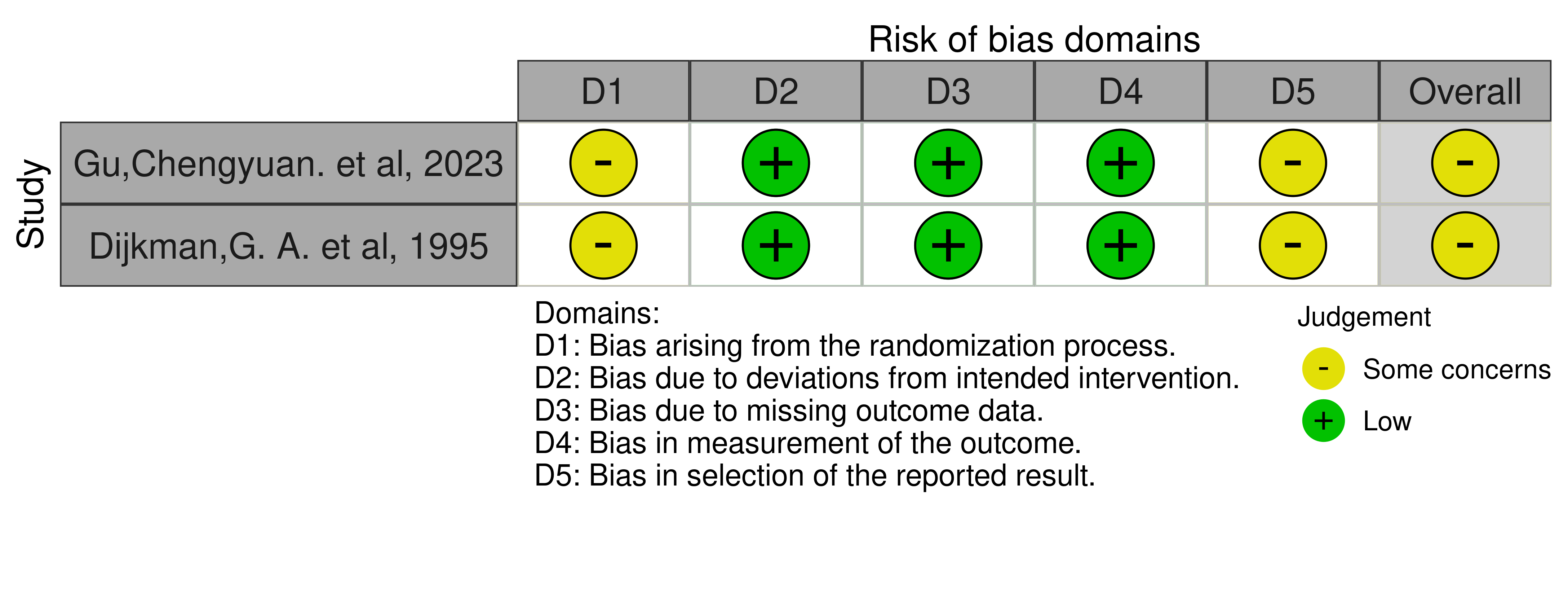

Supplement: Supplementary file 1 [file Image1.png]

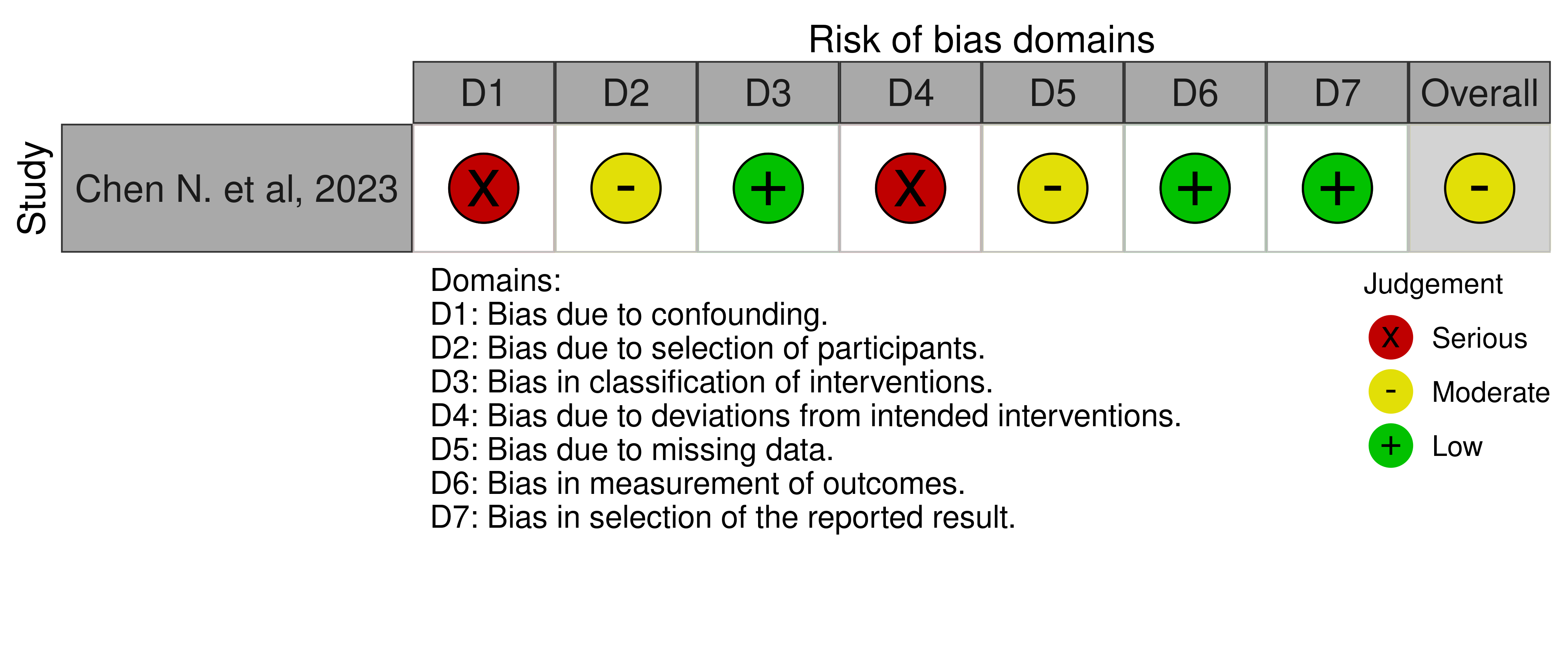

Supplement: Supplementary file 2 [file Image2.png]
