# Supplementary material for: Comparative effectiveness and integrated safety of goserelin sustained-release microspheres versus implants in prostate cancer: a patient-based real-world study and systematic review with meta-analysis
Source: Front Oncol. 2026 Jun 17;16:1858453. doi: 10.3389/fonc.2026.1858453 (PMC13318708; doi:10.3389/fonc.2026.1858453)
Supplement: Supplementary file 4 [file Table2.docx]

**Supplementary Material：** Detailed Search Strategy

The literature search was conducted in PubMed, Embase, Cochrane Library, and Web of Science from database inception to February 1,2026. Search strategies were adapted for each database according to its specific syntax and indexing system.

**1. PubMed**

#1 'neoplasms, prostatic' OR 'neoplasm, prostatic' OR 'prostatic neoplasm' OR 'prostate neoplasms' OR 'neoplasms, prostate' OR 'neoplasm, prostate' OR 'prostate neoplasm' OR 'prostate cancer' OR 'cancer, prostate' OR 'cancers, prostate' OR 'prostate cancers' OR 'cancer of prostate' OR 'cancer of the prostate' OR 'prostatic cancer' OR 'cancer, prostatic' OR 'cancers, prostatic' OR 'prostatic cancers'

#2 'goserelin' OR 'zoladex' OR 'ici-118630' OR 'ici118630' OR 'ici 118630' OR 'goserelin acetate' OR 'acetate, goserelin'

#3 #1 AND #2

**2. Embase**

#1 'neoplasms, prostatic' OR 'neoplasm, prostatic' OR 'prostatic neoplasm' OR 'prostate neoplasms' OR 'neoplasms, prostate' OR 'neoplasm, prostate' OR 'prostate neoplasm' OR 'prostate cancer' OR 'cancer, prostate' OR 'cancers, prostate' OR 'prostate cancers' OR 'cancer of prostate' OR 'cancer of the prostate' OR 'prostatic cancer' OR 'cancer, prostatic' OR 'cancers, prostatic' OR 'prostatic cancers'

#2 'goserelin' OR 'zoladex' OR 'ici-118630' OR 'ici118630' OR 'ici 118630' OR 'goserelin acetate' OR 'acetate, goserelin'

#3 #1 AND #2

**3. Cochrane Library**

#1 'neoplasms, prostatic' OR 'neoplasm, prostatic' OR 'prostatic neoplasm' OR 'prostate neoplasms' OR 'neoplasms, prostate' OR 'neoplasm, prostate' OR 'prostate neoplasm' OR 'prostate cancer' OR 'cancer, prostate' OR 'cancers, prostate' OR 'prostate cancers' OR 'cancer of prostate' OR 'cancer of the prostate' OR 'prostatic cancer' OR 'cancer, prostatic' OR 'cancers, prostatic' OR 'prostatic cancers'

#2 'goserelin' OR 'zoladex' OR 'ici-118630' OR 'ici118630' OR 'ici 118630' OR 'goserelin acetate' OR 'acetate, goserelin'

#3 #1 AND #2

**4. Web of Science**

#1 'neoplasms, prostatic' OR 'neoplasm, prostatic' OR 'prostatic neoplasm' OR 'prostate neoplasms' OR 'neoplasms, prostate' OR 'neoplasm, prostate' OR 'prostate neoplasm' OR 'prostate cancer' OR 'cancer, prostate' OR 'cancers, prostate' OR 'prostate cancers' OR 'cancer of prostate' OR 'cancer of the prostate' OR 'prostatic cancer' OR 'cancer, prostatic' OR 'cancers, prostatic' OR 'prostatic cancers'

#2 'goserelin' OR 'zoladex' OR 'ici-118630' OR 'ici118630' OR 'ici 118630' OR 'goserelin acetate' OR 'acetate, goserelin'

#3 #1 AND #2
